# Supplementary material for: GreenGate 2.0: Backwards compatible addons for assembly of complex transcriptional units and their stacking with GreenGate
Source: PLoS One. 2023 Sep 8;18(9):e0290097. doi: 10.1371/journal.pone.0290097 (PMC10490876; doi:10.1371/journal.pone.0290097)
Supplement: S1 Fig — Key components and cloning sites of the GreenBraid destination plasmids (pGB). The PaqCI/AarI and Eco31I recognition sites are depicted as arrows, and the corresponding overhangs are filled boxes with the overhang in lower letters. (PDF) [file pone.0290097.s001.pdf]

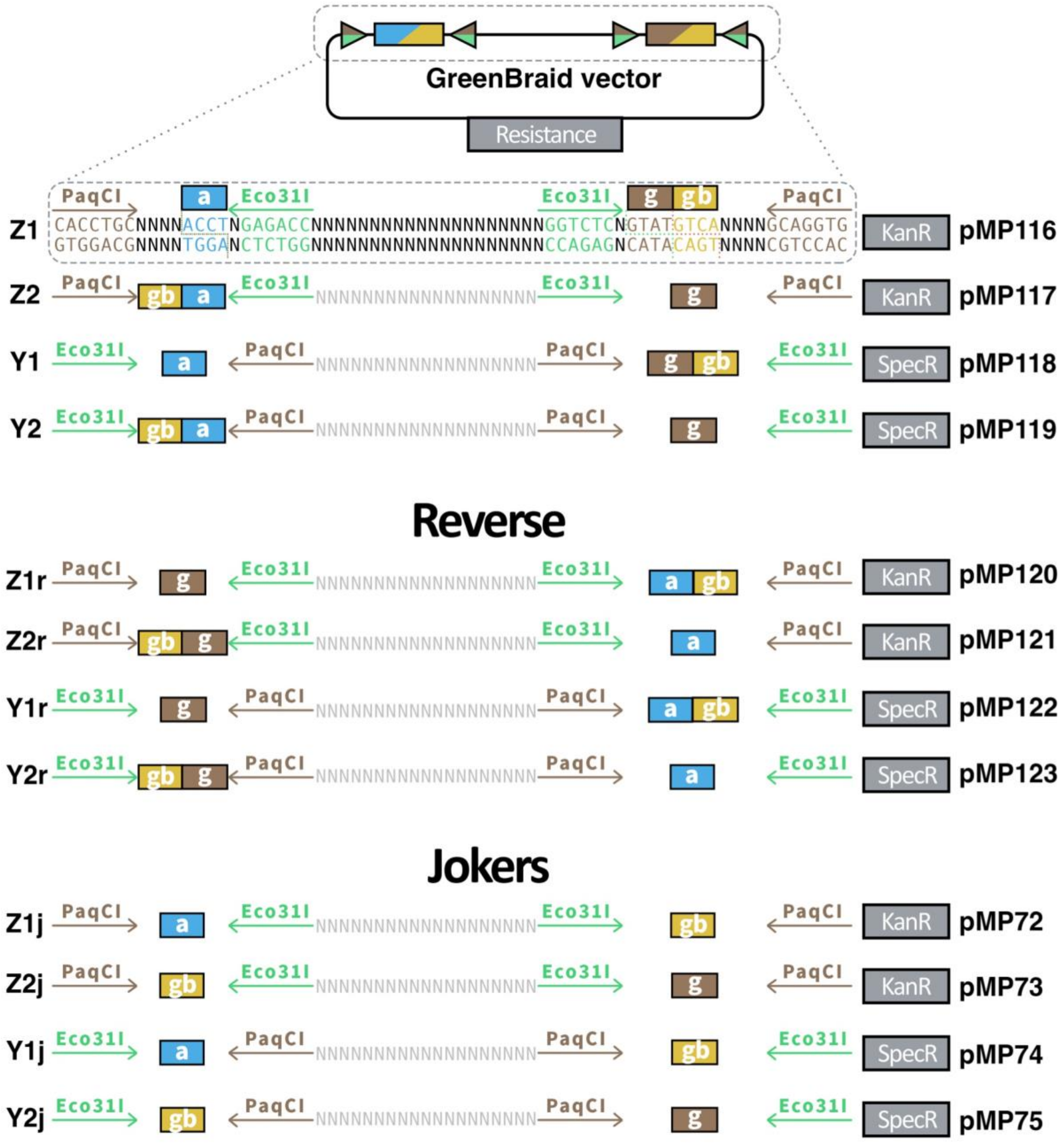

S1 Fig. Schematic overview of all GB destination plasmids and their name.

Key components and cloning sites of the GreenBraid destination plasmids (pGB). The PaqCI/AarI and Eco31I recognition sites are depicted as arrows, and the corresponding overhangs as filled boxes with the overhang in lower letters.
