# Supplementary material for: GreenGate 2.0: Backwards compatible addons for assembly of complex transcriptional units and their stacking with GreenGate
Source: PLoS One. 2023 Sep 8;18(9):e0290097. doi: 10.1371/journal.pone.0290097 (PMC10490876; doi:10.1371/journal.pone.0290097)
Supplement: S2 Fig — (PDF) [file pone.0290097.s002.pdf]

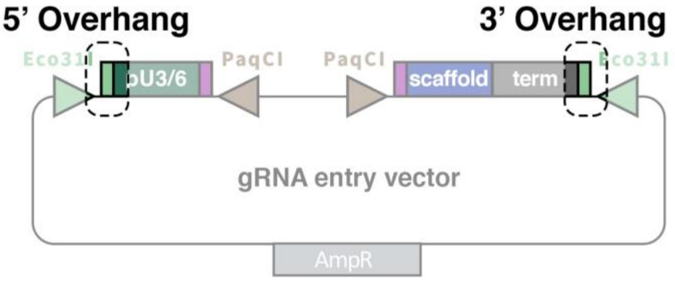

Name	Module	Promoter	5’ Overhang	3’ Overhang
pCS017	C1	pU6	GGCT	AGCC
pKE010	C2	pU3	AGCC	TTCG
pCS018	C3	pU6	TTCG	GCAG
pKE011	C4	pU3	GCAG	TCAG
pCS019	D1	pU6	TCAG	TGAC
pKE012	AD2	pU3	TGAC	TCCC
pCS020	D3	pU6	TCCC	CTGC
pKE013	E1	pU3	CTGC	CCAT
pCS021	E2	pU6	CCAT	ACTA

Fig S2. Schematic overview of all Level 0 plasmids for multiplex expression of gRNAs for CRISPR/Cas9 genome editing and their name.
