# Supplementary figures and images for: GreenGate 2.0: Backwards compatible addons for assembly of complex transcriptional units and their stacking with GreenGate
Source: PLoS One. 2023 Sep 8;18(9):e0290097. doi: 10.1371/journal.pone.0290097 (PMC10490876; doi:10.1371/journal.pone.0290097)

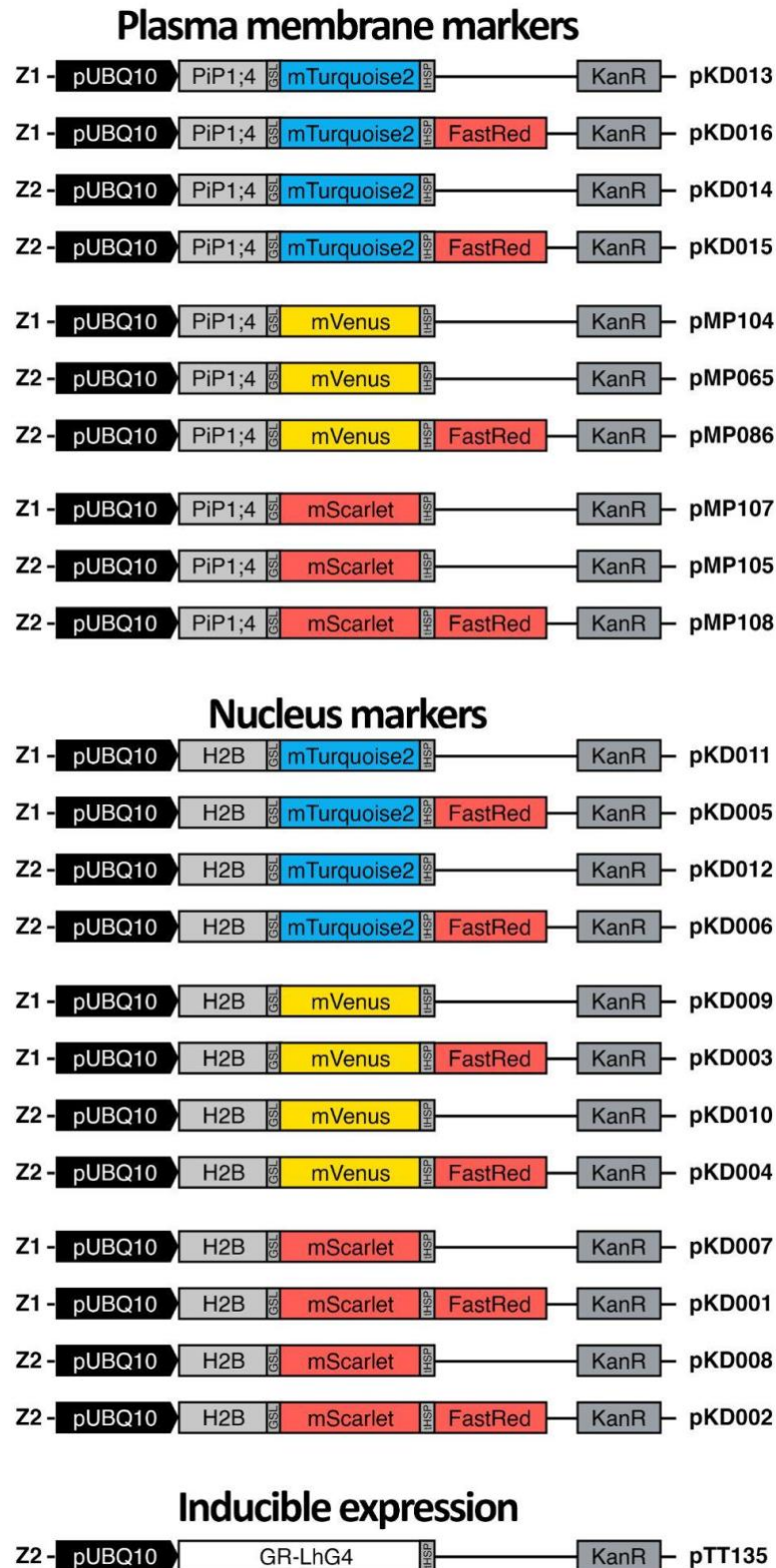

**Fig S3. Schematic overview of all filled GB destination plasmids and their name.**

Supplement: S3 Fig — (PDF) [file pone.0290097.s003.pdf]
