# Supplementary material for: GreenGate 2.0: Backwards compatible addons for assembly of complex transcriptional units and their stacking with GreenGate
Source: PLoS One. 2023 Sep 8;18(9):e0290097. doi: 10.1371/journal.pone.0290097 (PMC10490876; doi:10.1371/journal.pone.0290097)
Supplement: S1 Protocol — (PDF) [file pone.0290097.s007.pdf]

Supplemental Protocol: Creating entry GG clones and domestication using the pUEG

Designing primers for the generation of a Level 0 clone

Design a pair of primers according to the following template to amplify and clone an insert in pUEG:

Primer	Sequence (5' → 3')	Notes
**Forward**	ggCACCTGCaacaCTCA**xxxxNNNNNNNNNNNNNNNNNNNNNN**	**xxxx** → GreenGate 2.0 specific overhang (see table below)<br><br>**NNNNN** → Gene specific part (18-24 nt)
**Reverse**	ccCACCTGCtgttCTCG**xxxxNNNNNNNNNNNNNNNNNNNNNN**	

Module	5' overhang	3' overhang
**A**	ACCT	AACA
**B**	AACA	GGCT
**C**	GGCTcc	TCAG
**D**	TCAGgt	CTGC
**E**	CTGC	ACTA
**F**	ACTA	GTAT

**A1**	ACCT	TGAC
**AD2**	TGAC	TCCC
**A3**	TCCC	AACA
**A4**	TGAC	AACA
**A5**	ACCT	TCCC
**B1**	AACA	CCAT
**B2**	CCAT	GGCT
**C1**	GGCT	AGCC
**C2**	AGCC	TTCG
**C3**	TTCG	GCAG
**C4**	GCAG	TCAG
**C5**	AGCC	TCAG
**C6**	TTCG	TCAG
**C7**	GGCT	TTCG
**C8**	GGCT	GCAG
**D1**	TCAG	TGAC
**D3**	TCCC	CTGC
**D4**	TGAC	CTGC
**D5**	TCAG	TCCC

**E1**	CTGC	CCAT
**E2**	CCAT	ACTA
**I**	AACA	CTGC

Designing primers for the domestication of internal sites or assembly of several fragments

- Design four primers to obtain two amplicons to eliminate the illegal site and allow cloning in pUEG according to the following diagram:

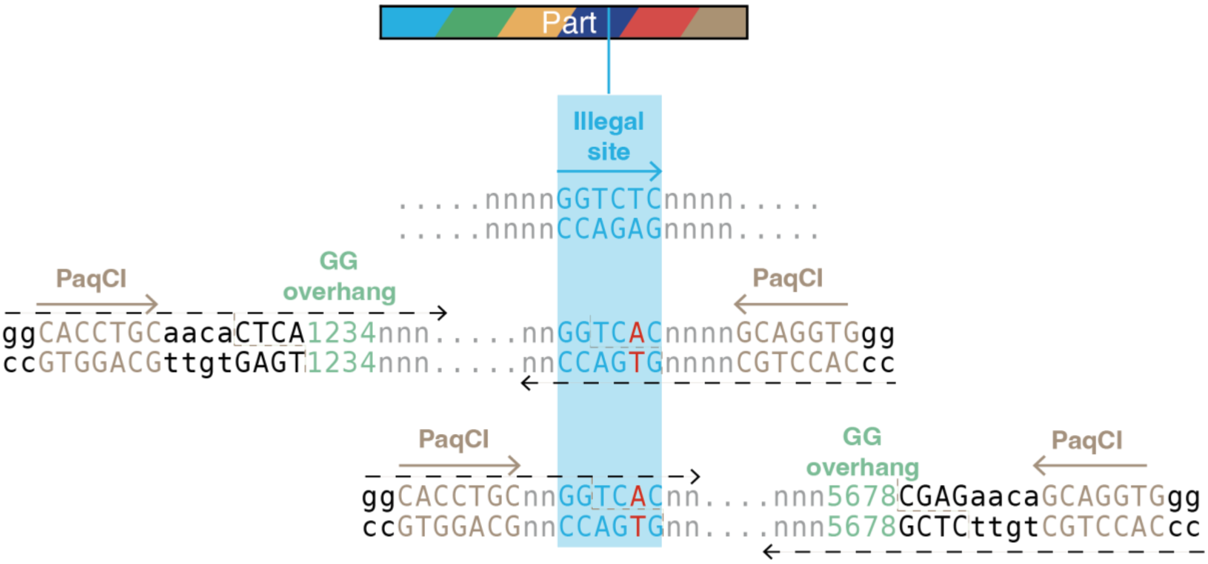

The dashed arrows are the primers to design.

`nnnnn` indicate the gene specific sequence

`1234`and `5678` are the GGv2.0 overhangs

Protocol for generation of a Level 0 clone

- Amplify your insert(s) by PCR using the primers designed above.
- Column or gel purify PCR products to get rid of free primers and/or enrich for bands of interest
- Set up the following GreenGate reaction (see https://international.neb.com/protocols/2021/01/11/golden-gate-assembly-protocol-using-paqci-neb-r0745-and-t4-dna-ligase-neb-m0202)

Component	Amount
pUEG plasmid	100 ng
10X T4 DNA Ligase buffer (B0202S)	2 µL
PaqCI Activator (20 µM)	1 µL
PaqCI (NEB R0745; 10 u/µl)	1 µL
T4 DNA Ligase (M0202, 400 u/µl)	1 µL
Insert(s)	2:1 molar ratio (insert(s) : vector backbone)
Nuclease-free H_2O	up to 20µL

- GreenGate assembly (on Thermocycler):

Temperature (°C)	Time (min:s)	Cycles
37	1:00	60x
16	1:00	
37	5:00	
60	5:00	
4	∞	

Time for protocol completion: ~2.5h. Assembly can be done in the morning, and transformation in *E.coli* can be done the same day. If the assembly is done overnight, add a 4°C terminal hold to
