# Supplementary material for: GreenGate 2.0: Backwards compatible addons for assembly of complex transcriptional units and their stacking with GreenGate
Source: PLoS One. 2023 Sep 8;18(9):e0290097. doi: 10.1371/journal.pone.0290097 (PMC10490876; doi:10.1371/journal.pone.0290097)
Supplement: S2 Protocol — (PDF) [file pone.0290097.s008.pdf]

Supplemental Protocol: GreenGate assembly of Level 0 modules into a Level 1 vector

Use Fast Digest/High Fidelity enzymes for GreenGate reactions and plasmids concentration (~100-200ng/µL). For assemblies of more than seven modules, use high concentrations of T4 DNA ligase (2000 u/µL).

1. Assemble the reaction in a PCR tube in the following order (top to bottom, enzymes last!)

**Component**		**Volume/ concentration**
entry clone A family	A/ A1-A5	1.5 µL
entry clone B family	B/ B1-B2	1.5 µL
entry clone C family	C/ C1-C8	1.5 µL
entry clone D family	D/ D1-D5	1.5 µL
entry clone E family	E/ E1-E2	1.5 µL
entry clone plant resistance	F	1.5 µL
destination vector	Z	1.0 µL
10x T4 ligase buffer		1x
CutSmart buffer		2.0 µL
H_2O		up to 30 µL
T4 DNA ligase (HC → 2000 u/µL)		1.0 µL
BsaI HFv2		1.0 µL

**TOTAL**		**30**

→ Do not use master mixes when you pipet the GG reaction. Most of the water in the reaction comes from the plasmids, which usually vary and are not part of the master mix you might kill the enzymes because of high salt concentrations before you even start.

2. Run the following program O/N on a PCR machine

**Temperature (°C)**	**Time (min:s)**	**Cycles**
37	5:00	50x
16	5:00	
50	5:00	
80	5:00	
4	∞	

3. The morning after, add 0.5 µL Eco31I and incubate at 37°C for 1h, then 80°C for 15 min to inactivate

4. Transform *E.coli* cells with 5 µL in chemically competent cells and 1 µL in electro-competent cells

5. Plate the *E.coli* on selective LB plates and incubate them O/N at 37 °C

6. Screen your colonies by colony PCR, and/or restriction digest for the correct assembly.
