# Supplementary material for: GreenGate 2.0: Backwards compatible addons for assembly of complex transcriptional units and their stacking with GreenGate
Source: PLoS One. 2023 Sep 8;18(9):e0290097. doi: 10.1371/journal.pone.0290097 (PMC10490876; doi:10.1371/journal.pone.0290097)
Supplement: S3 Protocol — (PDF) [file pone.0290097.s009.pdf]

Supplemental Protocol: GreenBraiding

The assembly of Level 2 plant transformation vector with multiple TUs can be divided into three steps:

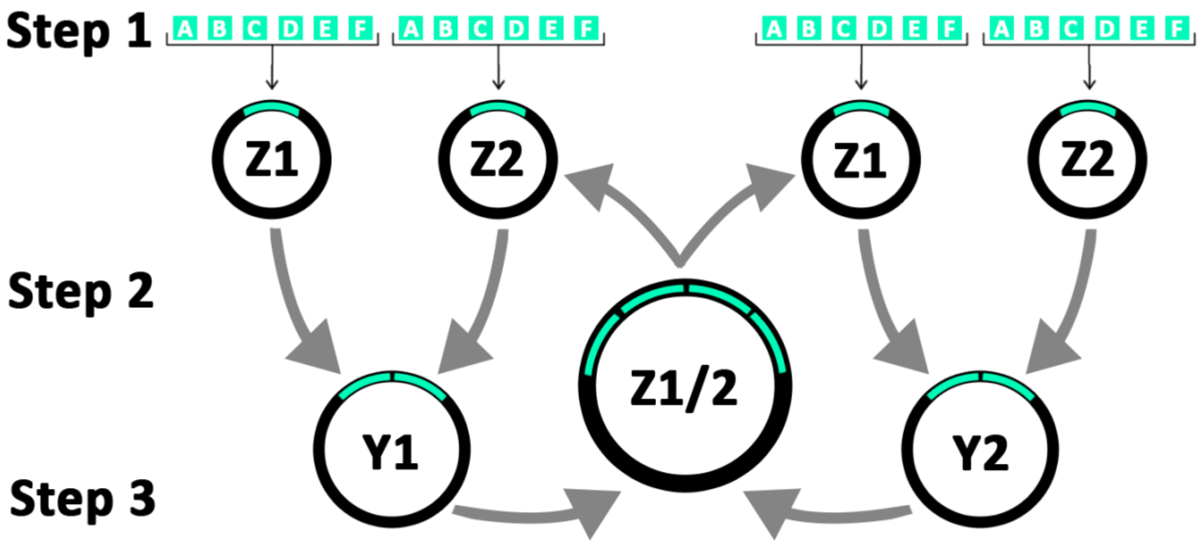

- **Step #1:** Create multiple level 1 vectors in parallel using level 0 entry and Z destination vectors.
- **Step #2:** Create level 2 vector(s) using level 1 Z vectors and Y destination vector(s).
- **Step #3:** Create a vector with more than two TUs by stacking level 2 Y vectors in a Z destination vector.

Table. **Materials used to assemble a four-gene construct.**

Product	Concentration	Volume	Provider	Package	Catalog name
Eco31I	1 µl/rxn	100 µl	ThermoFisher Scientific	FD0294	FD0294
FastDigest Buffer	10x	1 ml	ThermoFisher Scientific		
T4 DNA Ligase	30 U/ml		ThermoFisher Scientific	EL0013	EL0013

ATP	100 mM		ThermoFisher Scientific	R0441	
PaqCI	10.000 U/ml	0.02 ml	New England Biolabs	R0745S	R0745S
PaqCI activator	20 µM	0.02 ml	New England Biolabs	R0745S	S0532S
CutSmart buffer	10x	1.25 ml	New England Biolabs	R0745S	B7204S
T4 DNA ligase	400.000 U/ml	0.05 ml	New England Biolabs	M0202S	M0202S
T4 DNA ligase buffer	10x	0.5 ml	New England Biolabs	M0202S	B0202A

Step #1 Assemble individual transcriptional units

Step #1.1 Assembly

Assembly of multiple Level 1 vectors is done by GreenGate assembly in one of the **GreenBraid Z destination vectors (Table A & Table B):**

- Z1 / Z1r (reverse)

or

- Z2 / Z2r (reverse)

Important notes

- Pay attention to which vector you use to construct your TU; if you later want to combine two TUs, you can only combine Z1 (or Z1r) with Z2 (or Z2r).
- If you plan to combine several TUs on a single T-DNA only one of your TU should contain a plant selection marker and the others a dummy F clone (pMP059).

Table A: TU assembly in pGBZx mix. Components are listed in order of addition to the mix.

Component	Description	Amount	Volume (µl)
MilliQ Water			Up to 20
pGGA entry clone	Promoter	~100ng	1.5
pGGB entry clone	N-tag / linker	~100ng	1.5
pGGC entry clone	Gene coding sequence	~100ng	1.5
pGGD entry clone	C-tag / linker	~100ng	1.5
pGGE entry clone	Terminator	~100ng	1.5
pGGF entry clone	Plant selection marker*	~100ng	1.5
pGBZ destination vector<br>(pMP116 / pMP117 / pMP120 / pMP121)		~100ng	1.0
FastDigest buffer		1x	2.0
ATP		1 mM	2.0
Eco31I		0.5 rxn	0.5
T4 DNA Ligase (EL0013)		15 U	0.5

Table B: Gene assembly in pGBZ thermal cycling protocol.

Time for protocol completion: 8h50min.

Temperature (°C)	Time (min:s)	Cycles
37	5:00	50x
16	5:00	
50	5:00	
80	5:00	
4	∞	

Step #1.2 Assembly digest

Digest the assembly mix with Eco31I to minimize contamination with destination vectors that do not have an assembled TU in the next transformation Step #(**Table C**). Incubate the mix for 1h at 37°C and subsequently heat-inactivate Eco31I at 80°C for 15min.

Table C: post-assembly digestion mix.

Component	Amount
Assembly mix	20µl (all)
FastDigest buffer	~1x (2µl)
Eco31I	1 rxn

Step #1.3 Transformation in *E.coli*

Transform 5µl of the post-assembly treated mix into 100µl of chemically competent *E.coli* strain:

- Incubate on ice for 30min
- Heat shock for 30s at 42°C, without shaking
- Place on ice for 2min
- add 500µl of LB medium

- Recover for 1h in a shaking (225rpm) incubator at 37°C.
- Plate all cells (600µl) on LB+antibiotic resistance marker (pGBZ → kanamycin).

Step #1.4 Bacterial selection & inoculation

Pick colonies that do **not** have a green colour for mini-cultures (4ml LB + kanamycin). Although rare, the backbone of an entry clone may assemble in place of its module in the destination vector. To counterselect these, restreak picked colonies on LB plates+ampicillin and leave the plate O/N at 37˚C. GreenGate entry vector backbones carry an ampicillin resistance gene. Thus if a colony does **not** grow on this plate, it did not incorporate an entry vector backbone during assembly and can be further used in the next step.

Notes

- Typically we retrieve 100 to 600 colonies per plate.
- Selecting four colonies was typically sufficient to identify a clone that contains a plant transformation vector with an assembled TU.

Step #1.5 Screening for a vector with a correct assembly

To identify a clone that contains a vector with an assembly that incorporates all desired DNA fragments in the correct order, digest plasmids extracted from mini-cultures with one or several restriction enzymes that give a distinctive digestion pattern. We typically also verify by sequencing that the junctions between modules are correct.

Step #2 Combine two transcriptional units

Step #2.1 Combination assembly

Combine level 1 GreenBraid Z vectors (Z1 + Z2 exclusively) in a GreenBraid Y destination vector to create a level 2 vector:

- Y1 / Y1r (reverse)

  OR
- Y2 / Y2r (reverse)

Combine single TUs with a GreenBraid assembly#1 (**Table D & E**).

Important notes

- Pay attention to which vector you use to construct your TU; if you later want to combine TUs

housed in a Y, you can only combine Y1 (or Y1r) with Y2 (Y2r) in Step #3.

- If you want to create a T-DNA with an uneven amount of TUs or switch the backbone from a Z to a Y, then use a Z1j or Z2j as a dummy sequence.
- A GreenBraid assembly#1 is similar to a GreenGate assembly with a few alterations. Alterations are that a different restriction enzyme, PaqCI is used, that also requires the addition of PaqCI activation. A higher concentration T4 ligase (400U/µl) is also used alongside its buffer (Table D). Furthermore, the GreenBraid thermal cycling protocol is shorter than the GreenGate one.

Table D: GreenBraid assembly mix. Components are listed in order of addition to the mix. For the PaqCI activator it is wise to create an aliquot with concentration 5 pmol/µl from the stock. If a different buffer is used, ensure the final mix has 1mM ATP. C-dependent means concentration-dependent.

Component	Description	Amount	Volume (µl)
MilliQ Water			Up to 20
pGBZ1(r) entry vector<br>(pMP116 / pMP120)	Gene-of-interest 1	100ng	C-dependent
pGBZ2(r) entry vector<br>(pMP117 / pMP121)	Gene-of-interest 2	100ng	C-dependent
pGBY destination vector<br>(pMP118 / pMP119 / pMP122 / pMP123)		50ng	C-dependent
T4 DNA Ligase buffer (B0202S)	Has 10mM ATP	1x	2.0
PaqCI Activator		5pmol (1/4 of 20µM stock)	1.0
PaqCI		10 U	1.0
T4 DNA Ligase (M0202S)		400 U	1.0

Table: GreenBraid thermal cycling protocol. Time for protocol completion: 2h10min. If reactions are

done O/N, add a 4°C terminal hold to the protocol, but repeat the final 5 min heat-inactivation Step #at 60°C Step #the next day prior transformation.

Temperature (°C)	Time (min:s)	Cycles
37	1:00	60x
16	1:00	
37	5:00	
60	5:00	
4	∞	

Step #2.2. Assembly digest

Digest the assembly mix with PaqCI to minimise contamination with Y destination vectors that do not have an assembled TU in the next transformation Step #(**Table F**). Incubate the mix for 1h at 37°C and subsequently heat-inactivate PaqCI at 65°C for 20min.

Table F: post-assembly digestion mix.

Component	Amount
Assembly mix	20µl (all)
CutSmart Buffer	~1x (2µl)
PaqCI activator	5pmol
PaqCI	5 U

Step #2.3 Transformation in *E.coli*

Follow the same procedure as described in Step #1.3, except that recovered cells should be plated on

LB+spectinomycin.

Notes

- We saw plates with a few colonies (~10) and plates with many colonies (~600).

Step #2.4 Bacterial selection & inoculation

Follow the same procedure as described in Step #1.4, except that inoculation is done in LB+ antibiotic spectinomycin and counter-selection on plates with antibiotic kanamycin. For screening colonies that contain a vector with the desired assembly, picking four colonies is typically sufficient.

Step #2.5 Screen for vector with correct assembly

Follow the procedure as described in Step #1.5. In case sequencing of the plasmid is done, it may be sufficient to only sequence the regions at which ligation occurred rather than the entire plasmid.

Step #3 Assemble a construct with four transcriptional units

Step #3.1 Assembly

Combine level 2 GreenBraid Y vectors (Y1 + Y2 exclusively) in a GreenBraid Z destination vector to create another level 2 vector:

- Z1 / Z1r (reverse)

  OR
- Z2 / Z2r (reverse)

Combine doublets of TUs that are housed in Y1 and Y2 with a GreenBraid assembly#2 (**Table G & H**).

Important notes

- If you want to use the product of this assembly as input for Step #2, pay attention to which Z destination vector you use.
- If you want to create a T-DNA with an uneven amount of TUs or switch the backbone from a Y to a Z, then use a Y1j or Y2j as a dummy sequence.
- A GreenBraid assembly#2 is similar to a GreenGate assembly.

Table G: GreenBraid assembly#2 mix. Components are listed in order of addition. If a different buffer is used, ensure the final mix has 1mM ATP.

Component	Description	Amount	Volume (µl)
MilliQ Water			Up to 20
pGBY1(r) entry vector (pMP118 / pMP122)	Gene-of-interest doublet 1	150ng	C-dependent
pGBY2(r) entry vector (pMP119 / pMP123)	Gene-of-interest doublet 2	150ng	C-dependent
pGBZ destination vector (pMP116 / pMP117 / pMP120 / pMP121)		150ng	C-dependent
T4 DNA ligase buffer (B0202)	Contains 10mM ATP	1x	2.0
Eco31I		0.5 rxn	0.5
T4 DNA Ligase (M0202S)		400 U	1.0

Table H: GreenBraid assembly#2 thermal cycling protocol. Time for protocol completion 8h50min.

Temperature (°C)	Time (min:s)	Cycles
37	5:00	50x
16	5:00	
50	5:00	
80	5:00	
4	∞	

Step #3.2 Assembly digest

Follow the procedure described in Step #1.2.

Step #3.3 Transformation in *E.coli*

Follow the same procedure as described in Step #1.3.

Notes

- We typically obtain plates with ~50 colonies.

Step #3.4 Bacterial selection & inoculation

Follow the same procedure as described in Step #1.4, except that counter-selection is done on plates with antibiotic spectionomycin. For screening colonies that contain a vector with the desired assembly, picking four colonies is typically sufficient.

Step #3.5 Screen for vector with correct assembly

Follow the procedure as described in Step #1.5. In case sequencing of the plasmid is done, it may be sufficient only to sequence the regions at which ligation occurred rather than the entire plasmid.
